# Integrated Oral Microbiome and Metabolome Profiling Identifies Disease-Associated Multi-Omics Signatures in Alström and Bardet–Biedl Syndromes

**DOI:** 10.34133/csbj.0211

**Published:** 2026-09-03

**Authors:** Patrycja Mojsak, Ewa Zmyslowska-Polakowska, Sandra Chmielewska, Krzysztof Sołowiej, Tomasz Ploszaj, Sebastian Skoczylas, Julia Grzybowska-Adamowicz, Tomasz Pienkowski, Adam Kretowski, Agnieszka Zmyslowska, Michal Ciborowski

**Affiliations:** ^1^ Clinical Research Centre, Medical University of Bialystok, Bialystok, Poland.; ^2^ Department of Endodontics, Medical University of Lodz, Lodz, Poland.; ^3^ Department of Clinical Genetics, Medical University of Lodz, Lodz, Poland.; ^4^ Department of Endocrinology, Diabetology and Internal Medicine, Medical University of Bialystok, Bialystok, Poland.; ^5^ Department of Medical Biochemistry, Medical University of Bialystok, Bialystok, Poland.

## Abstract

**Background:** Alström syndrome (ALMS) and Bardet–Biedl syndrome (BBS) are rare ciliopathies characterized by multisystem involvement, including obesity, insulin resistance, and type 2 diabetes. Systemic metabolic dysfunction may influence the oral microbiome; however, integrative analyses that combine microbial and metabolic profiles in these disorders remain limited. **Methods:** Saliva and gingival crevicular fluid (GCF) samples were collected from genetically confirmed ALMS and BBS patients, as well as from obesity and healthy control groups. Microbial communities were profiled using V3–V4 16S rRNA gene amplicon sequencing, and untargeted metabolomic profiling was performed by gas chromatography–mass spectrometry. Microbiome–metabolome associations were evaluated using Spearman’s rank correlation analysis, followed by multi-omics integration using Multiple Co-Inertia Analysis (MCIA) and the supervised Data Integration Analysis for Biomarker discovery using Latent cOmponents (DIABLO) framework (mixOmics). **Results:** Integrated analysis identified distinct microbiome–metabolome association patterns in ALMS and BBS. Compared with controls, the ALMS+BBS group showed enrichment of *Prevotella*, *Enterococcus*, and *Eikenella*, alongside reduced *Lactobacillus* abundance. Metabolomic profiling revealed alterations in amino acid, fatty acid, and carbohydrate metabolism. GCF exhibited structured associations between metabolites and Firmicutes, Proteobacteria, and Actinobacteriota, whereas saliva showed broader interaction networks. These associations were absent or markedly weaker in obesity and healthy controls. MCIA demonstrated coordinated variation across the oral microbiome, salivary metabolome, and GCF metabolome, while DIABLO identified a shared multi-omics signature. **Conclusions:** Coordinated shifts in amino acid, lipid, and central carbon metabolism can be linked to oral microbial reorganization in ALMS and BBS. Integrative multi-omics analyses identified coordinated microbiome–metabolome signatures across the oral microbiome, saliva, and GCF. These findings warrant validation in larger longitudinal and functional studies.

## Highlights

• Integrated oral microbiome–metabolome profiling in ALMS/BBS
• Disease-specific oral microbial restructuring beyond obesity
• Distinct saliva and GCF microbiome–metabolome networks
• Organic, amino acid, and carbohydrate metabolism are associated with oral taxa
• Multi-omics reveals functional host–microbiome interactions

## Graphical Abstract

**Figure:**
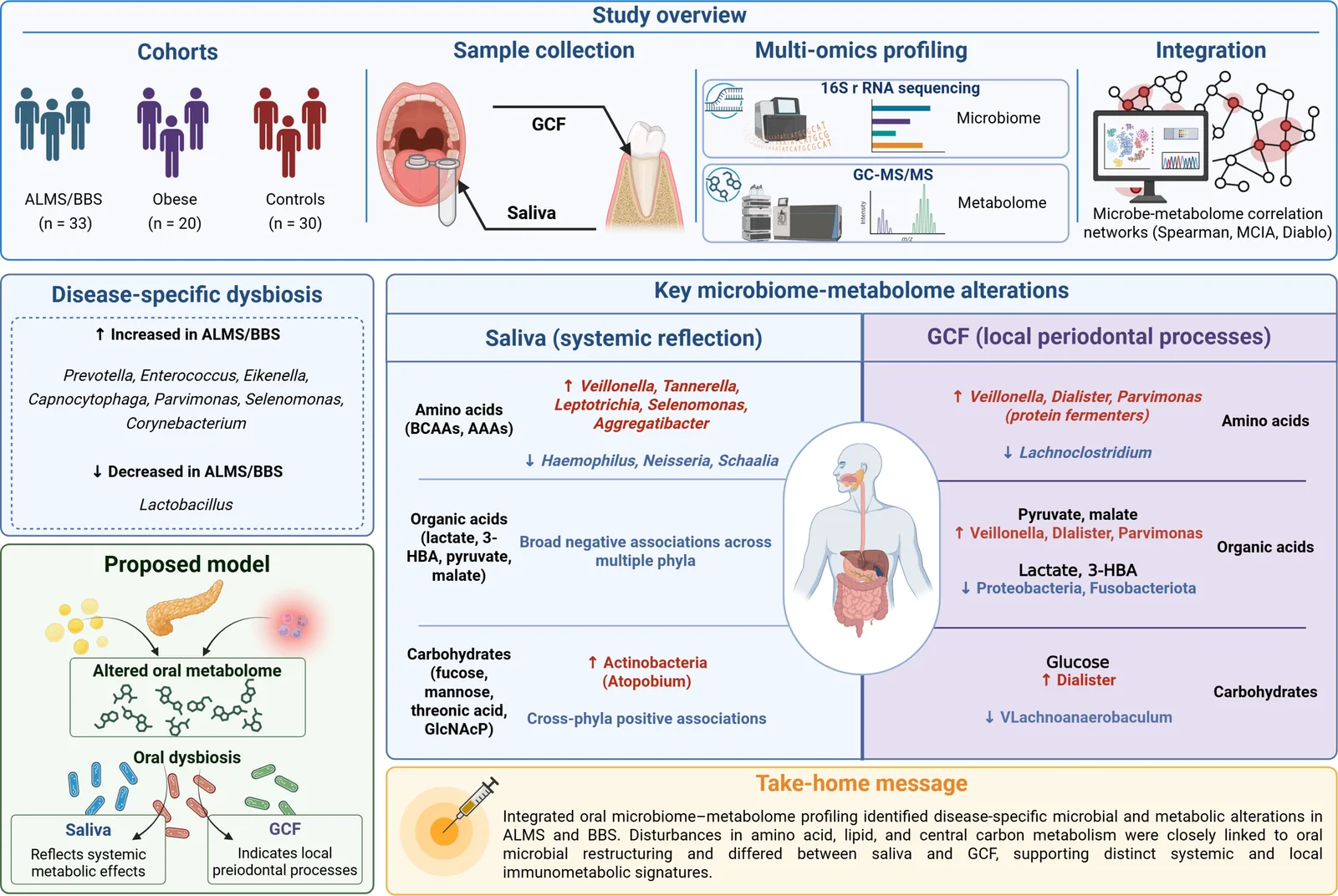

## Introduction

Alström syndrome (ALMS) and Bardet–Biedl syndrome (BBS) are rare inherited ciliopathies characterized by multisystem involvement, including progressive vision and hearing loss, metabolic dysfunction, and renal abnormalities [1]. These disorders result from mutations that disrupt the structure and function of primary cilia, leading to diverse phenotypes, such as obesity, insulin resistance, and retinal dystrophy [2]. The estimated prevalence of ALMS is around 1 in 1,000,000 individuals worldwide, with just over 1,200 cases reported globally [3]. By contrast, BBS is more common, affecting 1 in 125,000 to 160,000 live births, and certain populations exhibit founder effects that increase its incidence [4].

Obesity is an early clinical sign of both syndromes [5] and is often followed by insulin resistance and type 2 diabetes as metabolic complications develop [6,7]. Given the complex systemic effects of these ciliopathies, understanding changes in the oral microbiome is particularly important. However, studies directly analyzing the oral microbiome in ALMS and BBS remain limited [8]. Several oral bacterial taxa have been linked to metabolic dysregulation [9,10]. For example, members of Actinobacteriota, including *Actinomyces* and *Atopobium*, are reduced in type 2 diabetes [10], whereas gram-negative genera such as *Veillonella*, *Prevotella*, *Leptotrichia*, and *Catonella* have been associated with both diabetes and obesity [11,12]. Additionally, in overweight and obese individuals, oral microbial diversity often declines, with an overrepresentation of gram-negative taxa as body mass index (BMI) increases [13,14]. These alterations in the oral microbiome may promote inflammatory processes and exacerbate systemic metabolic disturbances [6,15].

In parallel, metabolomics offers a powerful means to elucidate biochemical changes in ALMS and BBS by profiling small-molecule metabolites in biological fluids such as saliva and gingival crevicular fluid (GCF). These syndromes involve complex metabolic dysfunctions, including insulin resistance, dyslipidemia, and renal impairment. Metabolomic profiling can identify disrupted metabolic pathways and molecular signatures associated with disease progression, potentially revealing diagnostic or prognostic biomarkers [16]. Importantly, integrating metabolomic and oral microbiome data may provide a more comprehensive understanding of coordinated microbiome–metabolome associations underlying disease-related metabolic alterations and facilitate the identification of multi-omics biomarkers and molecular signatures [17,18]. Despite this potential, no studies have explored this integrated approach in ALMS or BBS. Previous studies have demonstrated that the correlations between the oral microbiota and salivary/GCF metabolites are associated with dental caries, periodontitis, diabetes, and neurodegenerative diseases [19–21]. For example, GCF pathogens from the genus *Veillonella* showed positive correlations with pro-inflammatory metabolites, such as lactic acid and putrescine, in patients with type 2 diabetes mellitus (T2DM). In contrast, salivary microorganisms were more sensitive to changes in blood glucose levels, reflecting systemic metabolic alterations, and providing better diagnostic value for metabolic disruption in the patient organism [7]. Similarly, dysbiosis characterized by increased *Streptococcus* and *Veillonella* correlates with metabolites such as epinephrine and indoleacrylic acid, while acetate, propionate, and butyrate reflect inflammation and microbial activity in gingival tissues [22,23].

Despite growing interest in multi-omics approaches, the oral microbiome and metabolome in ALMS and BBS remain poorly characterized. To date, only our previous study has characterized the oral microbiome in this patient population, whereas existing metabolomic investigations have focused primarily on systemic biofluids, using serum liquid chromatography–quadrupole time-of-flight mass spectrometry (LC–QTOF-MS) and multi-matrix gas chromatography–mass spectrometry (GC–MS) [2,8,23]. Consequently, the oral cavity has not yet been investigated as an integrated source of microbial and metabolic biomarkers that reflect disease-associated alterations in ALMS and BBS, representing an important knowledge gap.

To address this gap, the present study combined oral microbiome profiling with untargeted metabolomic analysis of saliva and GCF. Disease-associated microbiome–metabolome relationships were first explored using pairwise Spearman correlation analysis and then examined using complementary multivariate multi-omics integration approaches, including Multiple Co-Inertia Analysis (MCIA) and the supervised Data Integration Analysis for Biomarker discovery using Latent cOmponents (DIABLO) framework. Unlike our previous studies, which analyzed the microbiome and metabolome separately, the present work integrates these complementary datasets to identify coordinated multi-omics signatures across the oral microbiome, salivary metabolome, and GCF metabolome. This integrative strategy provides a more comprehensive framework for investigating coordinated microbiome–metabolome variation in ALMS and BBS and generates hypotheses for future studies of ciliopathy-associated metabolic dysfunction.

## Materials and Methods

### Participants and study design

The study group comprised 10 participants from 10 families with genetically confirmed ALMS (*n* = 3) or BBS (*n* = 7), as previously described [2,8]. Additionally, 23 heterozygous carriers of pathogenic variants in ALMS1 (*n* = 10) or BBS-associated genes (*n* = 13) from the same families were included. Pathogenic variants in the BBS group were identified in BBS2, BBS8, BBS9, BBS10, and BBS12. Detailed demographic and clinical characteristics of all participants are summarized in Table 1 and have been described previously [2].

**Table 1.** Characteristics of the study groups

Parameters	Study group (patients with ALMS+BBS and heterozygotes)	Comparative group (patients with simple obesity)	Control group
	Mean ± SD	Mean ± SD	Mean ± SD
**Age at examination (years)**	34.2 ± 14.0	42.3 ± 14.4	30.1 ± 14.0
**BMI (kg/m** ^**2**^ **)**	34.3 ± 6.4	30.5 ± 3.9	21.3 ± 2.9
**Body weight (kg/m** ^**2**^ **)**	83.1 ± 24.3	88.1 ± 12.8	62.7 ± 10.2
***N* (F;M)**	33 (21 F + 12 M)	20 (13 F + 7 M)	30 (22 F + 8 M)

ALMS, Alström syndrome; BBS, Bardet–Biedl syndrome; BMI, body mass index; SD, standard deviation; F, female; M, male

All participants with ALMS or BBS were overweight or obese, while 14 of the 23 heterozygous carriers were overweight/obese and/or had type 2 diabetes. The comparison group comprised BMI-matched individuals with simple obesity, whereas healthy controls had normal BMI (<25 kg/m^2^) and no hyperglycemia, type 2 diabetes, retinal disorders, cardiomyopathy, renal dysfunction, or cirrhosis. Both the ALMS/BBS and comparison groups followed a low-glycemic-index, low-fat diet. Among the 33 participants in the ALMS/BBS group, 2 had type 2 diabetes and 2 had chronic kidney disease. None of the participants in any group had periodontal disease.

All participants underwent a comprehensive oral examination performed by 2 experienced dentists. Oral hygiene status was assessed using the Oral Hygiene Index (OHI) according to Greene and Vermillion, calculated as the sum of the plaque and calculus indices. The plaque index was scored from 0 to 3, where higher scores indicated increasing plaque and calculus accumulation.

Gingival inflammatory status was assessed using the Löe and Silness Gingival Index (GI) and the bleeding on probing percentage (BOP%) [24]. GI values did not exceed 0.2, and BOP% did not exceed 10% in any group. Moreover, no statistically significant differences were observed between the groups in GI (*P* = 0.41) or BOP% (*P* = 0.46). Subsequently, noninvasive samples were collected from the buccal mucosa and buccal gingival margin in the region of the first lower permanent molar from participants in the study, comparison, and control groups. The sampling method has been described as optimal and recommended for microbiome analyses, particularly in individuals without periodontitis [25,26]. Before sample collection, all participants were instructed to refrain from eating, smoking, and oral hygiene procedures (tooth brushing, flossing, and mouthwash use) for 12 h, and from drinking for 1 h before sampling. Samples intended for molecular analysis were collected into sterile screw-cap tubes and stored at −20 °C until further processing.

### Sample collection

Saliva samples were collected between 8:00 AM and 10:00 AM. All participants were instructed to refrain from eating, drinking, smoking, or performing oral hygiene for at least 8 h before sample collection. Saliva was collected using Salivette diagnostic collection tubes (Sarstedt, Germany) with synthetic swabs, which participants held in their mouths for 2 min. Samples were transported on ice immediately after collection and clarified by centrifugation at 4 °C (1,000 × *g* for 5 min). The supernatant was stored at −80 °C until metabolomic analysis.

Before collecting GCF, participants brushed their teeth in accordance with the provided instructions. GCF samples were then collected using sterile PERIOPAPER absorbent strips (Oraflow Inc., NY, USA), as described previously [8].

### Microbial DNA extraction and 16S rRNA gene sequencing

Detailed protocols for microbial DNA extraction, library preparation, sequencing, and bioinformatic processing were described previously [2]. Briefly, bacterial DNA was extracted from frozen buccal swab samples using the Maxwell RSC Pathogen Total Nucleic Acid Kit (Promega, Madison, WI, USA). DNA quality and concentration were assessed spectrophotometrically before amplification. The V3–V4 regions of the 16S rRNA gene were amplified, and libraries were prepared using Illumina Nextera indexing adapters. Sequencing was performed on the Illumina MiSeq platform (MiSeq Reagent Kit v3, 2 × 300 bp). Raw sequencing data were processed on the Galaxy platform, including read merging, quality filtering, and taxonomic assignment with Kraken2. Data were further filtered and normalized using total sum scaling (TSS), and relative abundances of bacterial taxa were calculated.

### Sample preparation for GC–MS analysis

Detailed protocols for untargeted metabolomic analysis of saliva and GCF have been described previously [2]. Briefly, metabolites were extracted from saliva and GCF samples and derivatized with O-methoxyamine hydrochloride and N-methyl-N-(trimethylsilyl)trifluoroacetamide (MSTFA) containing 1% trimethylchlorosilane (TMCS) prior to analysis. Quality control, extraction blank, and reagent blank samples were prepared and processed in parallel. Metabolic profiling was performed by GC–MS in full-scan mode. Raw data were processed by deconvolution, alignment, peak integration, and metabolite annotation using authenticated standards and reference libraries. Data preprocessing included filtering, missing-value imputation, and normalization before statistical analysis.

### Untargeted GC–MS data analysis

Metabolic fingerprinting was performed on a GC system (7890B series) equipped with a 7693A auto-sampler and a 7000D Mass Selective Detector (Agilent Technologies, Palo Alto, CA, USA). A 1-μl aliquot of the derivatized sample, including internal standards (ISs), was injected into a DB–5MS capillary GC column (30 m × 0.25 mm × 0.25 μm). Helium was used as the carrier gas at a constant flow rate of 1.0 ml/min. The injector temperature was maintained at 250 °C, with a split ratio of 10:1. The temperature program commenced at 60 °C with a 1-min hold, followed by a ramp to 320 °C at 10 °C/min. The GC–MS transfer line, filament source, and quadrupole were set at 280, 230, and 150 °C, respectively. The electron ionization source operated at 70 eV, and the mass spectrometer functioned in full-scan mode, covering *m*/*z* 50 to 600 at a scan rate of 1.38 scans per second.

### Raw GC–MS data processing

Deconvolution and compound identification were performed using Mass Hunter Quantitative Unknowns Analysis software (B.07.00, Agilent, Santa Clara, CA, USA). Data alignment was performed with Mass Profiler Professional software (version 13.0, Agilent, Santa Clara, CA, USA), and peak integration was conducted using Mass Hunter Quantitative Analysis software (version B.07.00, Agilent, Santa Clara, CA, USA). Compound identification relied primarily on accurate mass and product ion spectrum matching against an in-house library of authenticated standards, as well as reference libraries such as Fiehn’s and NIST 14. The complete list of annotated metabolites and their identification characteristics (including retention indices and spectral matching information) has been published previously and is available in our earlier study [2]; therefore, it is not reproduced here. Before statistical analysis, the areas of clinical sample peaks were normalized to minimize instrument-related response variability. For statistical comparisons, the GCF matrix was normalized to the total signal, while saliva and serum samples were normalized using the intensity of IS2.

### Statistical analysis

#### Spearman’s rank correlation

Before correlation analysis, metabolomics data were log-transformed to reduce skewness and stabilize variance. Microbiome data were treated as compositional and transformed using the centered log-ratio transformation. Associations between microbial taxa and metabolites were assessed using Spearman’s rank correlation and visualized with heatmaps and bubble plots. Statistical analyses and visualizations were conducted in R (version 4.5.2). Statistical significance was set at *P* < 0.05.

#### Multi-omics integration

To provide complementary perspectives on coordinated variation across the oral microbiome, salivary metabolome, and GCF metabolome, both unsupervised MCIA and supervised DIABLO analyses were performed.

#### Multiple co-inertia analysis

MCIA was performed in R (version 4.5.2) using the omicade4 package (version 1.50.0) as an unsupervised multivariate integration method to identify shared patterns of variation across multiple omics datasets without using group labels. Before integration, each dataset was preprocessed as described above, centered, and scaled. MCIA was then applied to generate a common multidimensional space that captures shared variation across the 3 omics layers. The contributions of individual datasets and molecular features to the identified components were examined to determine the main contributors to the shared multi-omics structure.

#### DIABLO multi-omics integration analysis

Supervised multi-omics integration was performed using DIABLO in the mixOmics R package (version 6.34.0). DIABLO identifies latent components that maximize covariance across omics datasets while discriminating predefined groups. Three data blocks, namely, the oral microbiome, salivary metabolome, and GCF metabolome, were integrated simultaneously. To prioritize coordinated relationships across datasets rather than classification performance, a moderate integration design matrix was used. A stronger connection was specified between the 2 metabolomic datasets (saliva–GCF = 0.3), whereas weaker connections were specified between the microbiome and each metabolomic dataset (0.1). Group separation based on the first 2 latent components was evaluated using permutational multivariate analysis of variance (PERMANOVA). Variable contributions to the first latent component were visualized with loading plots, and correlations among selected features across omics datasets were explored with Circos plots.

## Results

GC–MS-based metabolomic profiling of GCF, saliva, and serum samples from patients with ALMS and BBS, as well as control subjects, was reported previously [2]. Complementary oral microbiome analyses of the same cohort were published separately [8]. In the initial analyses, ALMS and BBS subgroups were evaluated separately. No statistically significant differences were identified between these groups in the metabolomic profiles of saliva or GCF, supporting their combined analysis as a single ALMS+BBS group in the present study [2]. Similarly, the comparison between homozygous individuals with ALMS/BBS syndromes and heterozygous carriers of pathogenic variants in ALMS1 and BBS-associated genes revealed no significant differences in oral hygiene status, assessed by the OHI (*P* = 0.32), or in microbial alpha diversity (*P* = 0.42). Therefore, these participants were combined into a single study group to increase statistical power, given the rarity of these genetic syndromes and the limited availability of affected individuals [8]. The microbiome study found that *Streptococcus* (30.7%), *Haemophilus* (18.9%), and *Prevotella* (11.0%) were the predominant bacterial genera across the cohort. Compared with controls, the ALMS+BBS group had higher abundances of *Prevotella*, *Enterococcus*, *Eikenella*, *Capnocytophaga*, *Parvimonas*, *Selenomonas*, and *Corynebacterium*, and a lower abundance of *Lactobacillus*. In addition, *Rothia*, *Schaalia*, and *Lactobacillus* showed the strongest discriminatory potential between the study groups [8]. Metabolomic analyses revealed significant alterations in 33 salivary and 19 GCF metabolites in the ALMS+BBS group compared with the obesity and control groups. Most of the altered metabolites were related to amino acid, fatty acid, and carbohydrate metabolism.

**Fig. 1.**
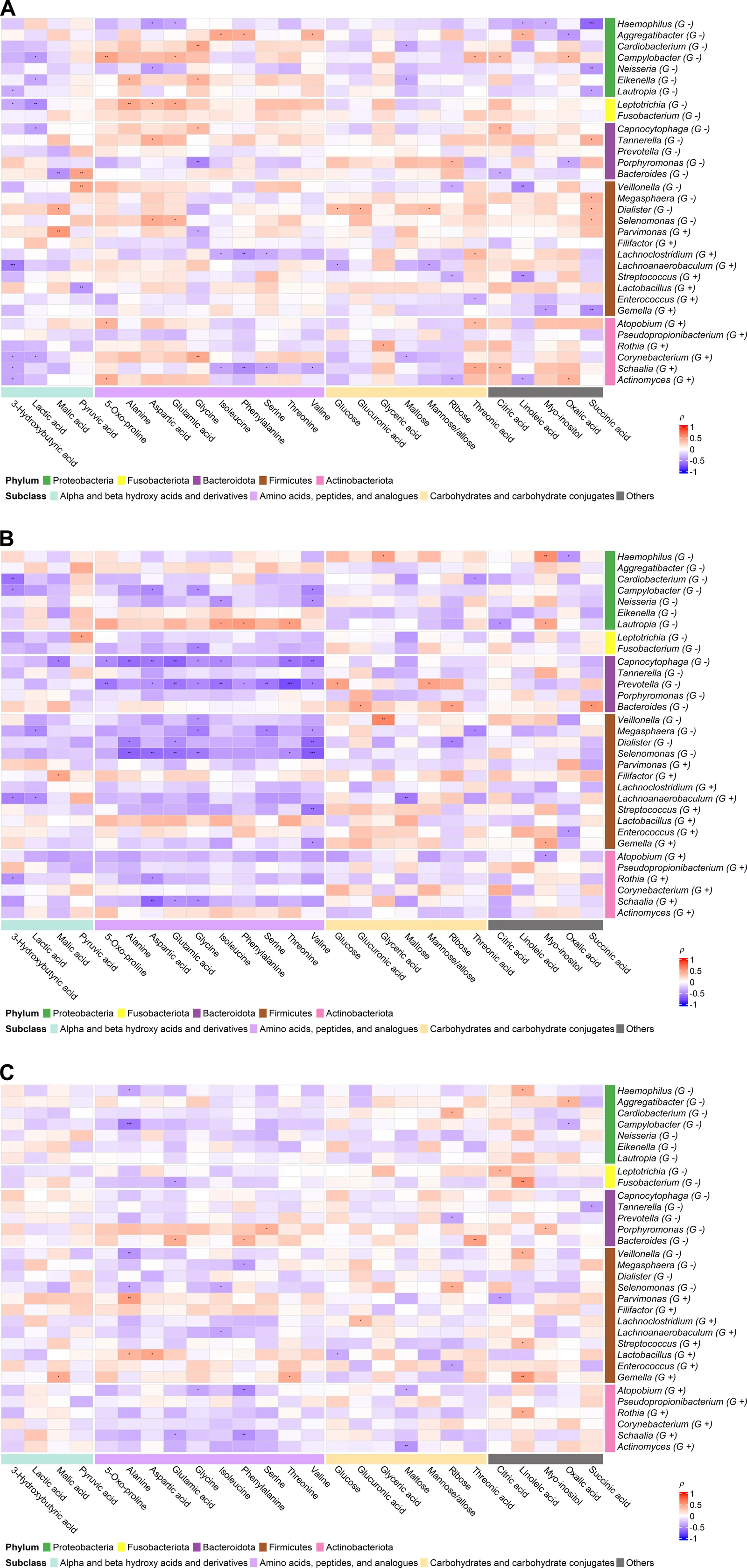
Correlation analysis between microbiome and metabolites for GCF samples in the study group (A), obesity (B), and control (C). Spearman’s rank correlation coefficients were calculated. Significance levels are indicated as follows: ***P < 0.001; **P < 0.01; *P < 0.05.

**Fig. 2.**
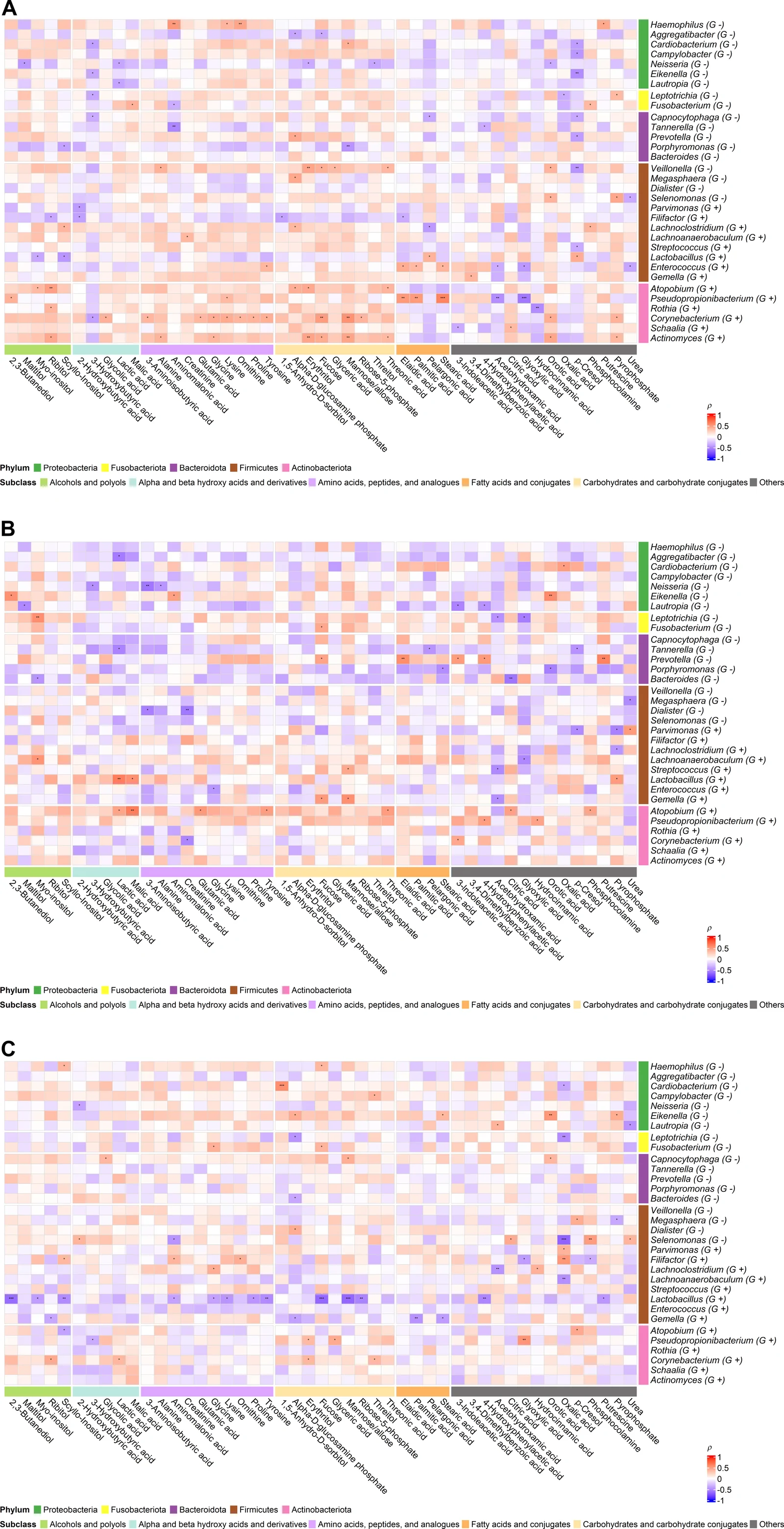
Correlation analysis between the microbiome and metabolites in saliva samples from the study group (A), obesity (B), and control (C). Spearman’s rank correlation coefficients were calculated. Significance levels are indicated as follows: ***P < 0.001; **P < 0.01; *P < 0.05.

**Fig. 3.**
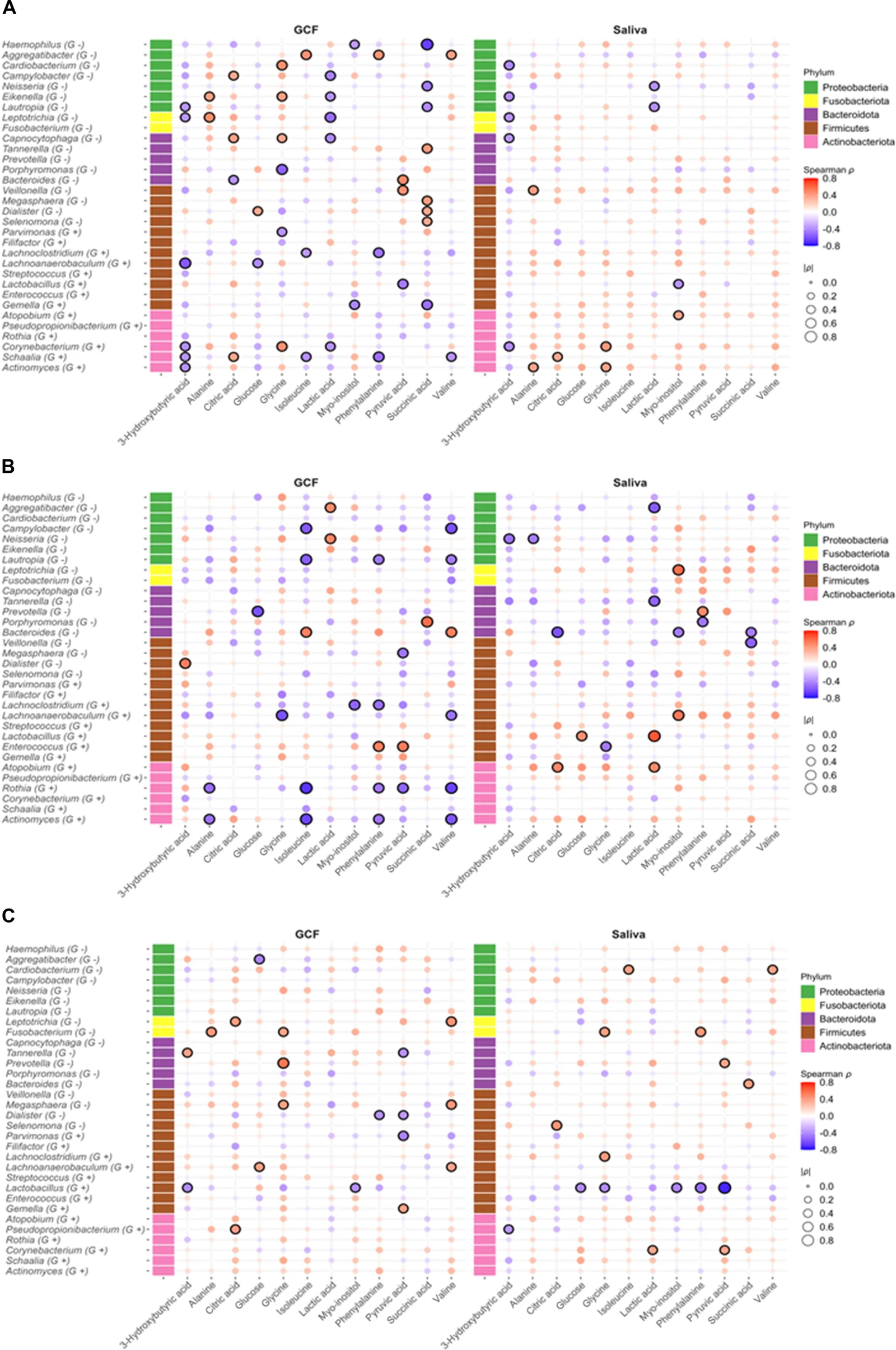
Bubble plots showing Spearman’s rank correlations between metabolites and 32 prevalent oral bacterial genera in the study group (A), obesity group (B), and control group (C). The left panel presents correlations in gingival crevicular fluid (GCF), whereas the right panel presents those in saliva. Bubble size indicates the strength of the correlation, while color denotes its direction (positive or negative). Statistically significant correlations (*P* < 0.05) are marked with a black border.

To examine associations between the oral microbiome and metabolomic profiles, Spearman’s rank correlations (*ρ*) were calculated separately for GCF (Fig. 1) and saliva (Fig. 2). The analysis included 32 prevalent bacterial genera, primarily from the phyla Actinobacteriota, Firmicutes, Proteobacteria, and Bacteroidota. Although all detected metabolites were included, Figs. 1 and 2 show only statistically significant associations, comprising 25 metabolites in GCF and 47 in saliva, mainly amino acids, fatty acids, and carbohydrates. The complete correlation matrices are provided in Tables S1 and S2. An overview of the statistically significant microbiome–metabolome associations is presented as bubble plots in Fig. 3, illustrating the direction and strength of the correlations in both biological matrices. Multiple positive and negative associations were observed, with statistically significant correlations highlighted by black outlines.

Only microbiome–metabolite correlations consistently observed in the ALMS+BBS group across both biological matrices were selected for detailed interpretation. The obesity and control groups served as reference cohorts to assess the specificity of these associations. Notably, the correlation patterns observed in the ALMS+BBS group were absent or markedly less consistent in the comparison groups, suggesting that these interactions may reflect disease-specific mechanisms associated with ALMS and BBS.

In the obesity group, a distinct correlation pattern was observed, characterized by strong negative correlations between amino acid metabolites in GCF and representatives of the Bacteroidota phylum, particularly the gram-negative genera *Capnocytophaga* and *Prevotella*, as well as selected gram-negative Firmicutes, including *Selenomonas*, *Dialister*, and *Megasphaera*. These relationships were absent in the other groups.

### Gingival crevicular fluid

In the study group, Spearman’s correlation analysis of GCF metabolites and oral microbiome composition revealed distinct patterns of association across metabolite classes and bacterial taxa (Fig. 1). Negative correlations were predominantly observed between the levels of alpha- and beta-hydroxy acid derivatives and the abundance of gram-positive representatives of the Firmicutes and Actinobacteriota phyla, as well as gram-negative Proteobacteria. In contrast, positive correlations were more frequently observed with amino acids and carbohydrate metabolites, as well as with members of Actinobacteriota, Proteobacteria, and gram-negative Firmicutes.

The levels of organic acids, including lactic acid and 3-hydroxybutyric acid (3-HBA), were negatively correlated with selected Proteobacteria genera, namely, *Lautropia*, *Eikenella*, and *Campylobacter*, as well as with the Fusobacteriota genus—*Leptotrichia*. In addition, 3-HBA showed multiple negative correlations with members of Actinobacteriota and a strong negative correlation with *Lachnoanaerobaculum* (Firmicutes). By contrast, malic acid and pyruvic acid levels were positively correlated with the abundance of gram-negative Firmicutes, including *Veillonella* and *Dialister*.

The levels of amino acid metabolites, including isoleucine, phenylalanine, serine, and valine, showed predominantly negative correlations with the abundance of gram-positive Firmicutes (e.g., *Lachnoclostridium*) and Actinobacteriota (e.g., *Schaalia*), whereas positive correlations were observed primarily with Proteobacteria. Similarly, glycine, glutamic acid, and alanine were positively correlated with Proteobacteria and Bacteroidota.

Glucose levels were positively correlated with the abundance of *Dialister* and negatively correlated with *Lachnoanaerobaculum*, despite both genera belonging to the Firmicutes phylum. Carbohydrate metabolites showed more heterogeneous association patterns, with both positive and negative correlations involving representatives of Proteobacteria, Firmicutes, and Actinobacteriota, including associations with threonic acid, mannose, maltose, and ribose levels.

Furthermore, succinic acid levels showed strong negative correlations with selected Proteobacteria genera, including *Haemophilus*, *Neisseria*, and *Lautropia*, whereas positive correlations were observed with the abundance of anaerobic Firmicutes, including *Megasphaera*, *Dialister*, and *Selenomonas*.

### Saliva

Analysis of the correlation between saliva metabolites and the oral microbiome revealed a more complex, less homogeneous pattern of relationships than in GCF, with a predominance of positive correlations across many metabolite classes.

Positive correlations were observed between the levels of several amino acids (tyrosine, proline, ornithine, lysine, glycine, and glutamic acid) and the presence of Actinobacteriota, particularly the gram-positive genus *Corynebacterium.* Additionally, associations were observed between Proteobacteria, including the genus *Haemophilus*, and the presence of ornithine, lysine, and aminomalonic acid.

Correlation analysis of metabolites identified in saliva and the microbiome revealed numerous negative associations, particularly involving p-cresol. The levels of this metabolite showed negative correlations with gram-negative bacteria belonging to the Proteobacteria phylum (including *Cardiobacterium*, *Campylobacter*, and *Eikenella*) and Bacteroidota (e.g., *Capnocytophaga* and *Prevotella*), as well as with selected representatives of the Firmicutes phylum, such as *Veillonella* and *Streptococcus*.

Correlations were observed between metabolites in the α- and β-hydroxy acid groups and their derivatives, including numerous negative correlations for 3-HBA levels, across representatives of various bacterial phyla: Actinobacteriota (*Corynebacterium*), Bacteroidota (*Capnocytophaga*), Fusobacteriota (*Leptotrichia*), and Proteobacteria (*Cardiobacterium*, *Eikenella*). For 2-hydroxybutyrate (2-HBA), correlations were observed with bacteria belonging to the Firmicutes phylum, including *Parvimonas* and *Filifactor*.

The levels of fatty acid metabolites (stearic acid, elaidic acid, and palmitic acid) showed a positive correlation with a member of the Actinobacteriota phylum (*Pseudopropionibacterium*, gram-positive) and a member of the Firmicutes phylum (*Enterococcus*, gram-positive).

A positive correlation was observed between the levels of carbohydrate metabolites and their derivatives, such as fucose, mannose, and threonic acid, and the presence of Actinobacteriota. The level of alpha-D-glucosamine phosphate was positively correlated not only with Actinobacteriota (*Atopobium*) but also with Firmicutes (*Megasphaera* and *Lachnoclostridium*) and Bacteroidota (*Prevotella*).

### Multiple co-inertia analysis

**Fig. 4.**
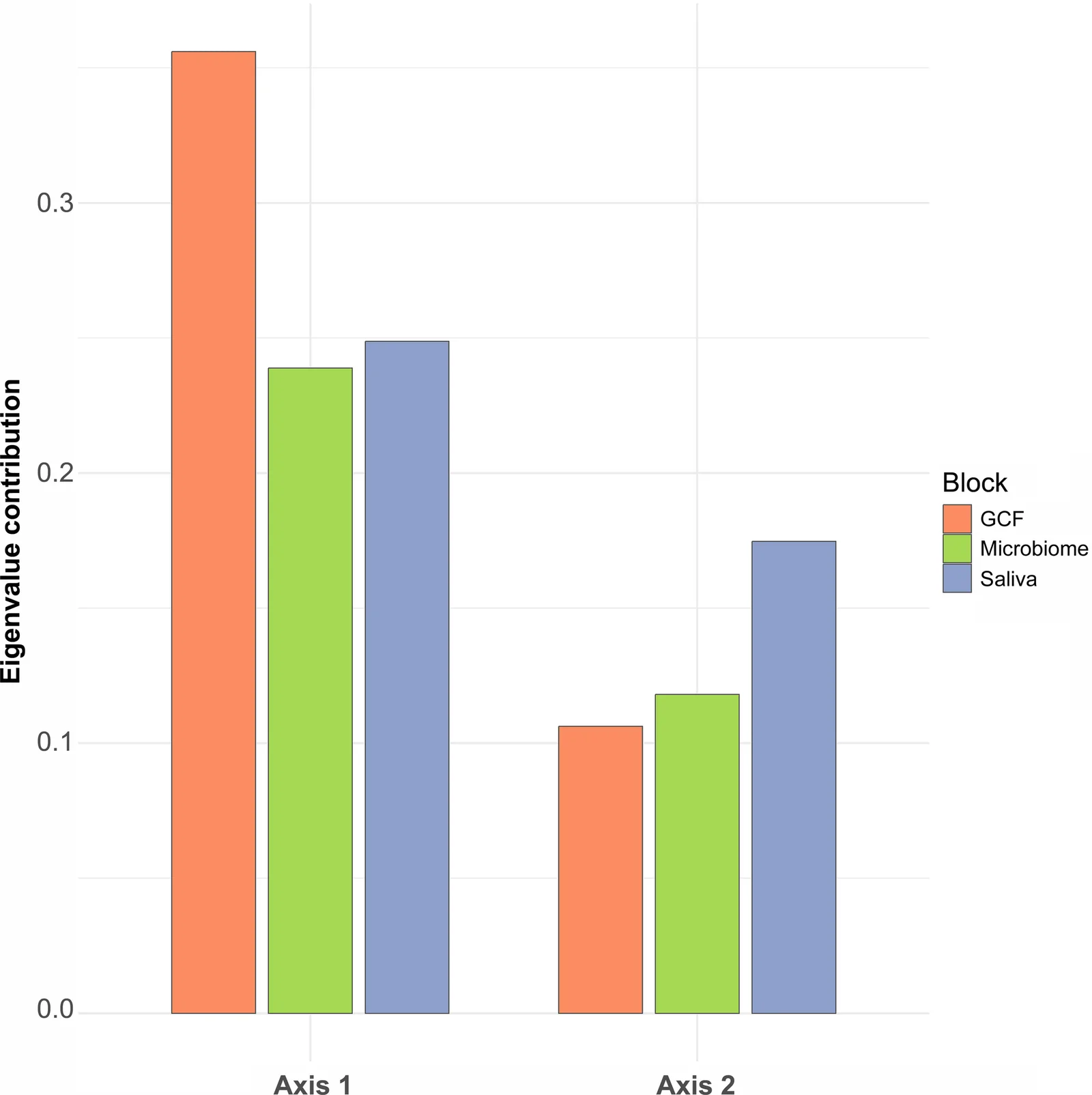
Relative contributions of the GCF metabolome, salivary metabolome, and oral microbiome to the first 2 MCIA components.

**Fig. 5.**
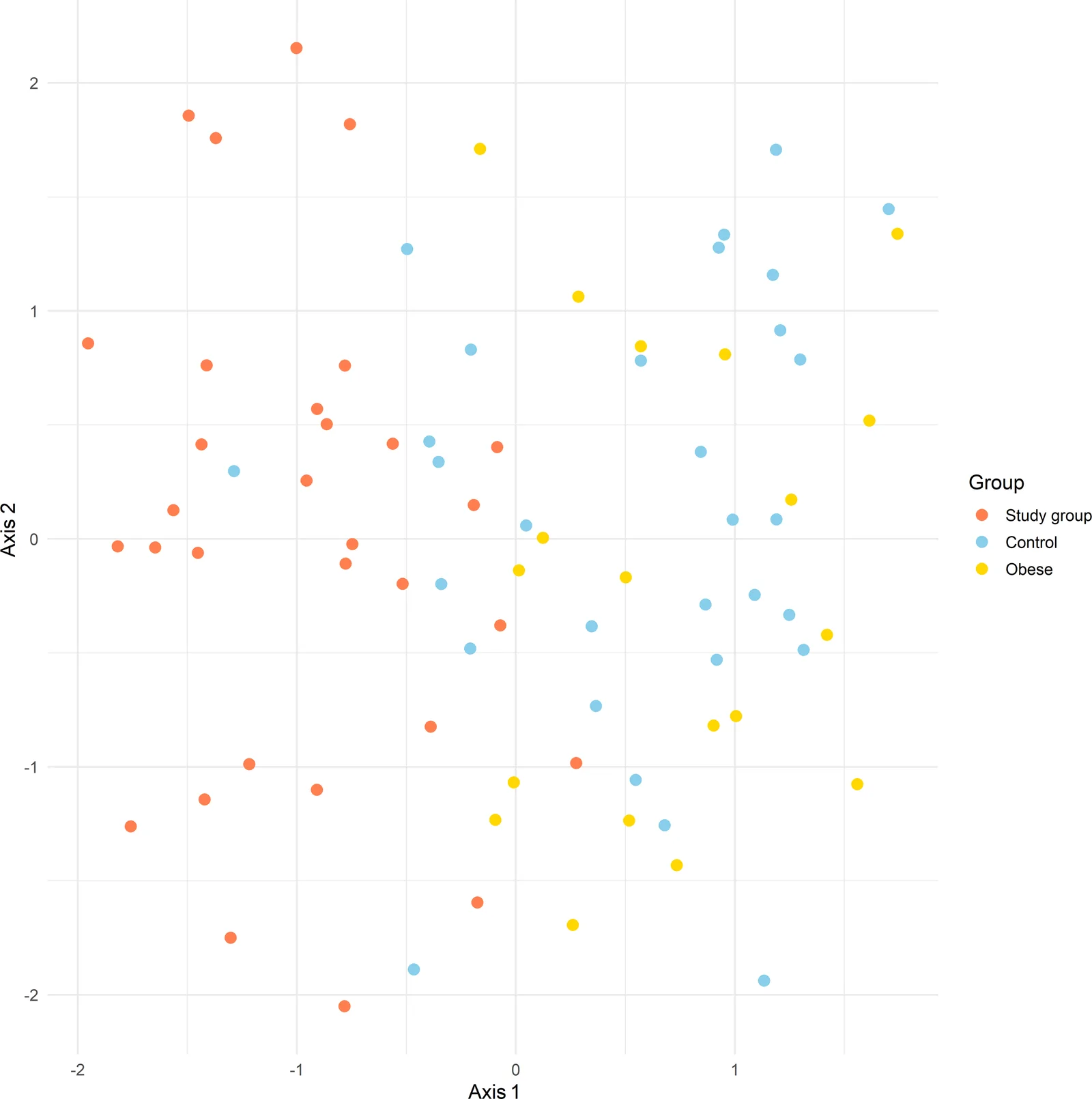
MCIA sample ordination. Projection of samples onto the first 2 MCIA components showing partial separation of the study groups and shared multi-omics variation across the integrated datasets.

To further investigate the global covariance structure across the GCF metabolome, salivary metabolome, and oral microbiome datasets, MCIA was performed. The first 2 MCIA components accounted for approximately 42% of the cumulative inertia across the integrated datasets (Fig. S1). The first MCIA component received contributions from all 3 omics layers, with the GCF metabolome contributing most strongly, whereas the second component was influenced predominantly by the salivary metabolome (Fig. 4). Projection of samples onto the first 2 MCIA components showed partial separation of the study groups, with substantial overlap, indicating coordinated variation across the integrated omics datasets despite considerable biological heterogeneity (Fig. 5). The variables contributing most strongly to the first and second MCIA components are presented in Fig. S2.

### Multi-omics integration using DIABLO

**Fig. 6.**
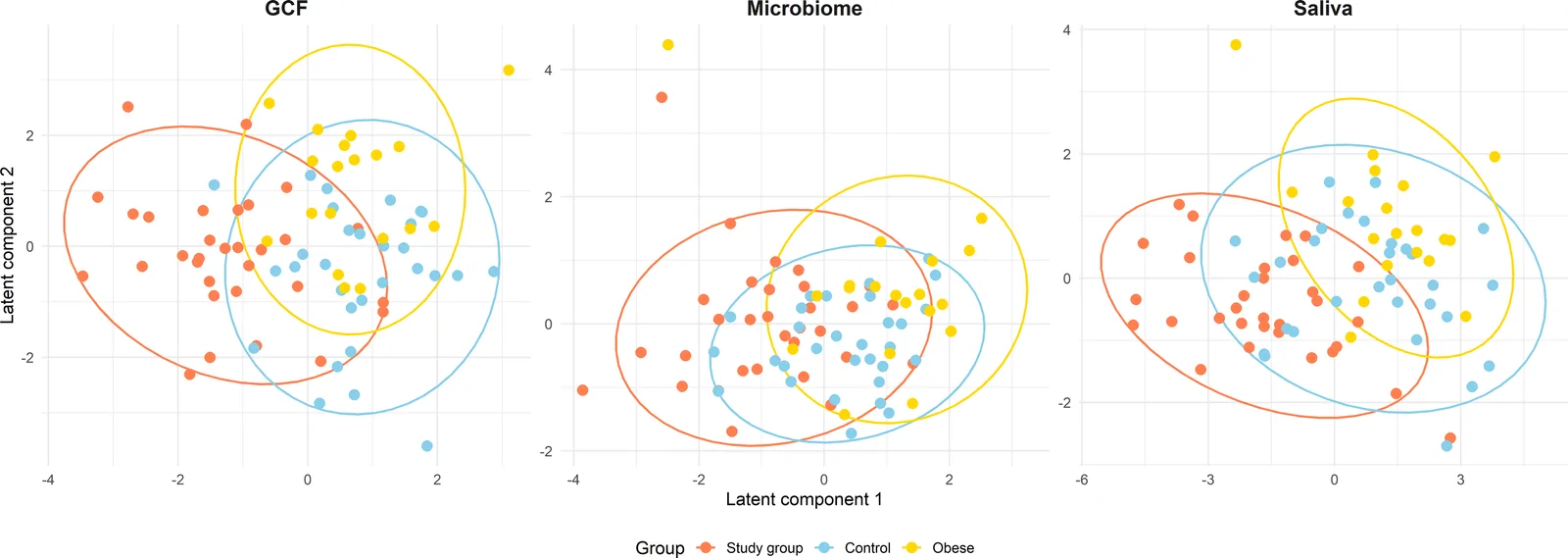
DIABLO sample projection onto the first 2 latent components across the GCF metabolome, salivary metabolome, and oral microbiome datasets. Each panel represents one omics block. Ellipses indicate the 95% confidence intervals for each study group.

To complement the pairwise correlation analyses, multi-omics integration was performed using DIABLO to identify coordinated patterns across the oral microbiome, salivary metabolome, and GCF metabolome. The model identified a common latent component shared across all 3 omics layers. Projection of samples onto the first 2 latent components showed partial separation of the study groups across the microbiome, salivary and GCF metabolome datasets (Fig. 6).

The discriminatory performance of the integrated model was supported by PERMANOVA, which confirmed significant differences between the study groups across all 3 data blocks (microbiome: *R*^2^ = 0.250, *P* < 0.001; saliva: *R*^2^ = 0.355, *P* < 0.001; GCF: *R*^2^ = 0.366, *P* < 0.001) (Table S1).

**Fig. 7.**
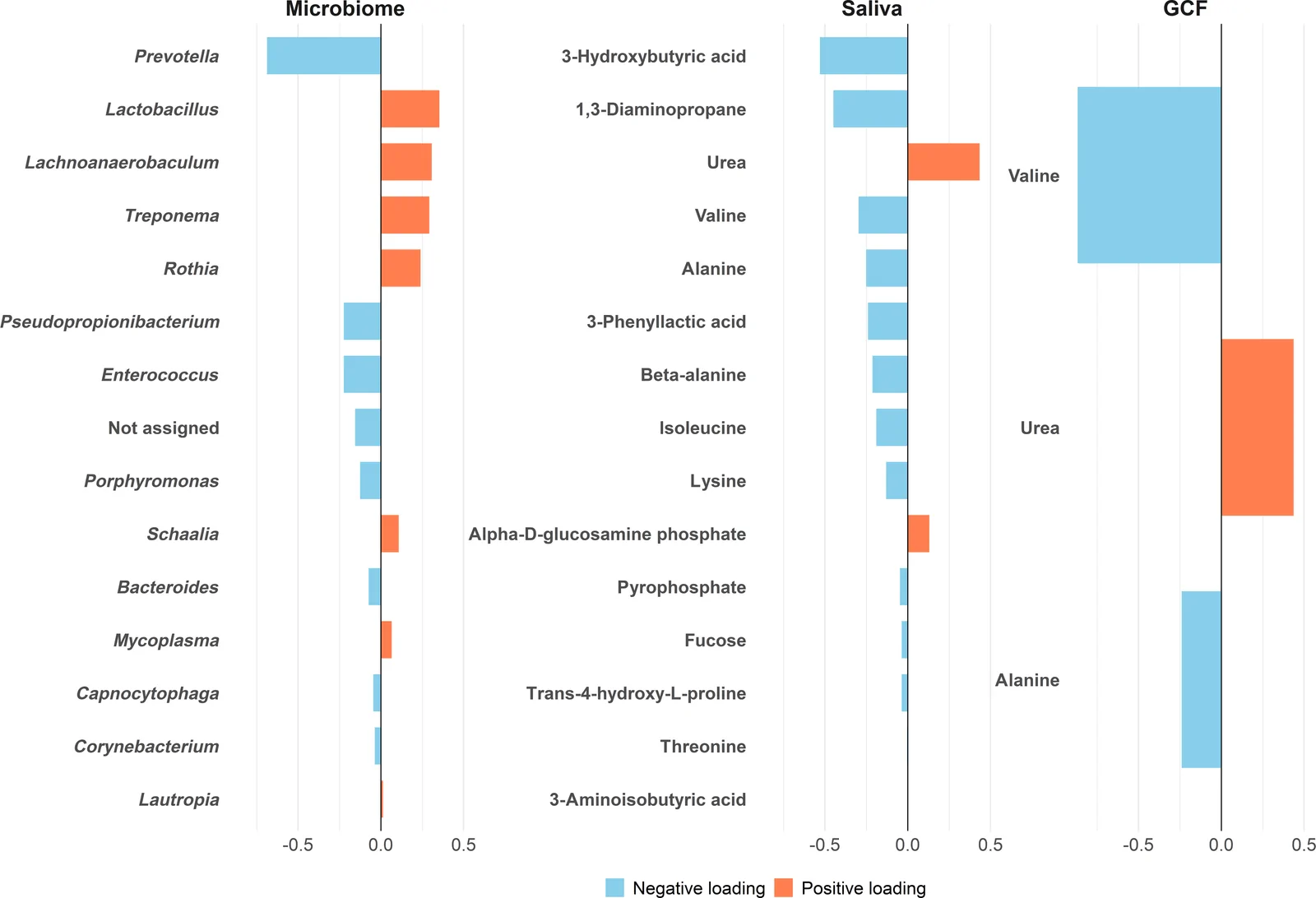
Variables with the highest absolute loadings on the first DIABLO latent component across the oral microbiome, salivary metabolome, and GCF metabolome datasets. Positive (orange) and negative (blue) bars indicate the direction and magnitude of variable loadings contributing to the integrated multi-omics signature.

**Fig. 8.**
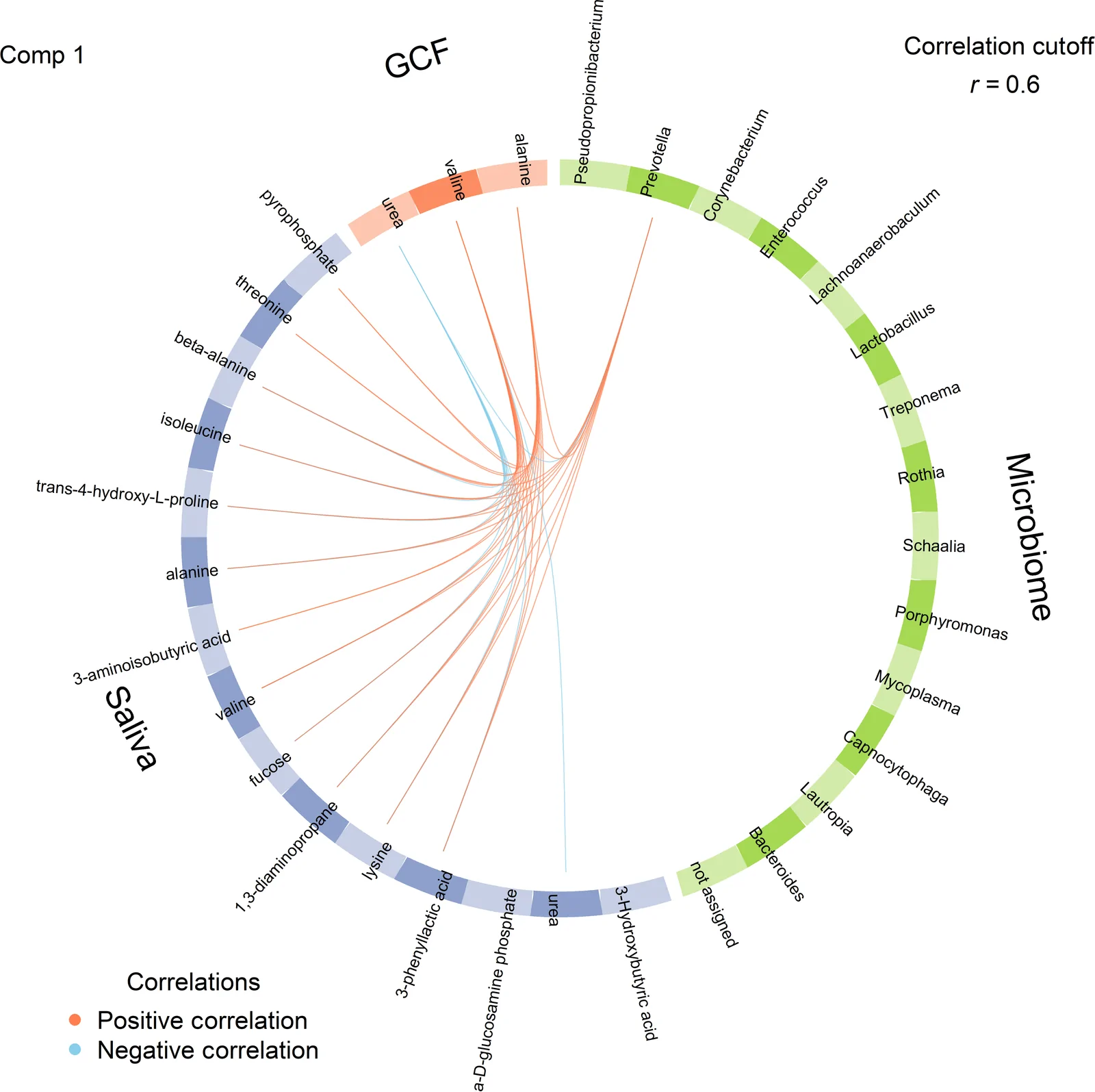
DIABLO Circos plot illustrating correlations among the variables selected by the first latent component across the oral microbiome, salivary metabolome, and GCF metabolome datasets. Red and blue lines represent positive and negative correlations, respectively (|*r*| ≥ 0.6). The outer profiles indicate the relative abundance or intensity of each feature across the study groups.

The variables with the highest absolute loadings on the first DIABLO latent component are presented in Fig. 7. The variables contributing most strongly to the first latent component included *Prevotella*, *Enterococcus*, *Lachnoanaerobaculum*, and *Lactobacillus* within the microbiome. In the salivary metabolome, the variables with the highest absolute loadings included 3-HBA, urea, valine, alanine, and α-D-glucosamine phosphate, whereas urea, valine, and alanine showed the highest absolute loadings in the GCF metabolome. Correlations among the selected features are illustrated in the DIABLO Circos plot (Fig. 8). The Circos plot revealed coordinated positive and negative associations between microbial taxa and metabolites across the salivary and GCF metabolomes, indicating coordinated variation among the 3 omics layers. These findings support the presence of coordinated microbiome–metabolome signatures across the integrated omics datasets.

The robustness of the selected features was further supported by repeated 80% subsampling, which demonstrated high selection frequencies for the principal microbial taxa and metabolites contributing to the integrated signature (Fig. S3).

## Discussion

This study is the first to explore links between the oral microbiome and metabolomic profiles in saliva and GCF in patients with ALMS and BBS. Saliva and GCF, though biologically distinct, reflect systemic and local processes. Saliva supports oral health and immunity, while GCF contains mediators involved in immune responses and periodontal protection. Both fluids are noninvasive and increasingly studied as biomarkers for metabolic and inflammatory diseases [27]. Evidence shows that microbial communities influence host metabolism, contributing to obesity [28], insulin resistance [29], and T2DM [30] through interactions with metabolic and inflammatory pathways. Although most mechanistic insights come from gut microbiome studies, they support the hypothesis that host–microbiome–metabolome interactions contribute to metabolic disease, including ciliopathies such as ALMS and BBS, which are associated with profound metabolic disturbances. Because ALMS and BBS are characterized by disturbances in energy and systemic metabolism [2], exploring microbiome–metabolome associations may offer additional insights into disease-related metabolic alterations. Integrating oral microbiome and metabolomic analyses with integrative multi-omics approaches may enhance understanding of the metabolic alterations associated with ciliary dysfunction and identify coordinated disease-specific molecular signatures, without necessarily establishing direct mechanisms. However, such integrative analyses do not, by themselves, establish causal or mechanistic relationships.

Saliva and GCF showed disease-specific microbiome–metabolome correlations absent in controls and obese groups (see Fig. 3). This compartment-specific organization suggests that the 2 oral biofluids capture partially distinct dimensions of host–microbiome associations, with saliva potentially reflecting broader metabolic integration and GCF preserving greater sensitivity to local immunometabolic conditions. The dominant bacteria in the population were *Streptococcus* (30%), *Haemophilus* (18.9%), and *Prevotella* (11%), consistent with a previous report [8]. The ALMS/BBS group displayed higher levels of *Prevotella*, *Enterococcus*, *Eikenella*, *Capnocytophaga*, *Parvimonas*, *Selenomonas*, and *Corynebacterium*, and lower *levels of Lactobacillus*. The increased abundance of *Prevotella*, *Selenomonas*, and selected anaerobic taxa, together with reduced *Lactobacillus*, partially overlaps with microbial alterations previously reported in obesity and metabolic disorders [5,31]. However, inconsistent findings for taxa such as *Capnocytophaga* and *Corynebacterium* suggest that ALMS/BBS may exhibit a microbial configuration extending beyond patterns typically associated with obesity alone [31]. Although the present study combined pairwise correlation analyses with supervised multi-omics integration, these observations should be interpreted as reflecting coordinated biological associations rather than direct functional effects of individual taxa. The enrichment of these taxa may therefore suggest broader ecological and metabolic alterations extending beyond differences in body mass alone. The decreased abundance of *Lactobacillus* may reflect a reduction in taxa that previously associated with microbial homeostasis. Although evidence on *Lactobacillus*’ role in oral phenotypes is mixed, reduced beneficial bacteria often accompany dysbiosis and inflammation [32]. Shannon alpha diversity analysis showed no significant differences between groups, though the control tended to have higher microbial diversity [8]. In contrast, beta diversity analysis revealed significant differences in bacterial community composition between the ALMS/BBS group and other groups (*P* < 0.031), indicating that microbial patterns are influenced by factors beyond obesity and may be associated with ciliopathy-related metabolic disturbances [8]. The lack of differences in alpha diversity and the separation in beta diversity suggest that changes are mainly in community structure and specific taxa, not in total microbial richness [33]. These results support the presence of a unique oral microbial signature associated with disease-related metabolic and inflammatory alterations, beyond those associated with obesity.

This study highlights distinct microbiome–metabolome associations in saliva and GCF. Saliva exhibits a broader, positively correlated network that may reflect integration with systemic metabolic signals, whereas GCF displays structured, directional patterns consistent with stronger local immunometabolic regulation and greater sensitivity to microenvironmental factors such as inflammation and oxygen availability. This compartment-specific organization suggests that saliva and GCF capture partially distinct dimensions of host–microbiome associations rather than serving as interchangeable biological matrices. These findings are consistent with previous research showing that saliva reflects systemic status, whereas GCF reflects local immunometabolic processes, and GCF metabolomics has been associated with periodontal inflammation and microbial activity [34–36]. To complement the pairwise correlation analyses, we performed MCIA analysis, which revealed moderate shared covariance between the oral microbiome and metabolomic datasets. This indicates that part of the variability was shared across the omics layers, supporting coordinated cross-omics variation in the study cohort. These findings complement the pairwise Spearman correlations by indicating that the observed associations are not limited to isolated metabolite–taxon pairs, consistent with previous applications of co-inertia-based integration approaches [37].

Within these compartment-specific networks, amino acid–microbiome associations were among the most consistent patterns observed, particularly in GCF (not in saliva), involving branched-chain amino acids (BCAAs; valine and isoleucine) and aromatic amino acids (AAAs) (phenylalanine). Negative associations between these metabolites and representatives of Firmicutes (e.g., *Lachnoclostridium*) and Actinobacteriota (e.g., *Schaalia*), together with positive associations with Proteobacteria (e.g., *Aggregatibacter*), may reflect altered microbial ecological organization and differences in substrate availability across oral niches. Similar metabolic shifts toward proteolytic activity and amino acid fermentation have previously been associated with periodontal inflammation and dysbiotic oral biofilms [38]. Importantly, elevated concentrations of BCAAs and AAAs in saliva and GCF have previously been linked to insulin resistance and impaired metabolic flexibility [39]. In our study, metabolite levels were increased by 85% to 207% relative to the control and obese groups [2], suggesting that these alterations may not be attributable to obesity alone but may also reflect disease-specific metabolic dysregulation in ALMS and BBS. Interestingly, these associations were specific to GCF and were not observed in saliva, supporting the hypothesis that salivary metabolomic signatures may reflect broader systemic metabolic alterations, whereas GCF may be more sensitive to local periodontal conditions. Because altered insulin signaling, disrupted energy homeostasis, and systemic metabolic dysfunction are recognized features of both ALMS and BBS, the observed amino acid profile may reflect disease-associated metabolic remodeling rather than an isolated oral phenomenon. Notably, ALMS and BBS are characterized by profound metabolic disturbances, chronic low-grade inflammation, and altered energy homeostasis, all of which may shape host–microbiome associations within the oral environment [8].

Beyond amino acid metabolism, organic and hydroxy acid metabolites also exhibited distinct compartment-specific association patterns (see Figs. 1 and 2). In GCF, lactate and 3-HBA were negatively associated with several Proteobacteria and Fusobacteriota, suggesting that these metabolites may be linked to ecological conditions that differ from those favoring the enrichment of selected gram-negative taxa, rather than directly shaping microbial composition. By contrast, positive associations between pyruvate and malate and anaerobic genera such as *Veillonella*, *Dialister*, and *Parvimonas* may reflect differences in metabolite availability, cross-feeding, and cooperative metabolic networks within subgingival biofilm communities. These associations are consistent with previously described metabolic cooperation within oral biofilms, particularly involving lactate-utilizing anaerobes such as *Veillonella* [40], but do not directly demonstrate such interactions. These observations may also reflect ecological adaptation to local physicochemical conditions within periodontal biofilms, including oxygen availability, redox gradients, and nutrient flux, which are recognized contributors to subgingival microbial community organization and metabolic activity [41]. In saliva, hydroxy acids, particularly 3-HBA, showed broader, more diffuse negative associations across multiple bacterial phyla, potentially reflecting integration with systemic metabolic signals rather than solely local biofilm-related processes. This interpretation may be especially relevant in the context of systemic metabolic disturbances characteristic of ALMS and BBS, both of which are associated with profound alterations in lipid and energy metabolism. Altered levels of ketone body-related metabolites, including 3-HBA and other hydroxy acid intermediates, have been reported in metabolomic studies of ALMS/BBS patients [2], supporting the possibility that salivary metabolite profiles may partially reflect systemic metabolic dysregulation in addition to local oral biofilm-associated processes.

Many metabolites detected in saliva and GCF may originate from both host and microbial pathways; therefore, their biological origin cannot be determined from the present data. Accordingly, the observed associations likely reflect coordinated host–microbiome–metabolome interactions rather than taxon-specific production. Selective links between 2-HBA and Firmicutes suggest metabolite-specific associations. Broader salivary associations may indicate that saliva integrates oral and systemic signals. These findings suggest that organic acid metabolism may be associated with oral microbiome ecology, reflecting local and systemic disturbances characteristic of ALMS and BBS. Carbohydrate metabolism shows distinct patterns in GCF and saliva: glucose shows different associations with Firmicutes, suggesting varied ecological niches. Associations of threonic acid, mannose, maltose, and ribose with microbes are context-dependent, potentially influenced by host substrates and biofilm ecology. Salivary carbohydrate metabolites correlate with Actinobacteriota, potentially reflecting processes associated with biofilm formation. Alpha-D-glucosamine phosphate shows positive correlations across many phyla, suggesting coordinated responses across microbial taxa. These patterns further support the hypothesis that salivary metabolites may serve as biomarkers of host–microbiome associations and may also contribute to the ecological environment of oral biofilms. However, the present data do not allow determination of whether these metabolites originate from host or microbial metabolism, nor do they demonstrate their functional role within oral biofilms. Collectively, these findings highlight the importance of integrative multi-omics approaches for understanding coordinated host–microbiome associations that extend beyond taxonomic composition alone [38].

These observations were further supported by MCIA, which revealed a shared covariance structure across the oral microbiome, salivary metabolome, and GCF metabolome. MCIA is an unsupervised integration approach that identifies covariance shared across multiple omics datasets without using group labels. It therefore provides complementary evidence of coordinated cross-omics variation alongside the supervised DIABLO analysis. To further characterize these coordinated microbiome–metabolome associations, we applied DIABLO, a supervised multi-omics integration framework. Together, MCIA and DIABLO provided complementary evidence of coordinated cross-omics variation. MCIA demonstrated a shared covariance structure across the oral microbiome, salivary metabolome, and GCF metabolome, whereas DIABLO identified an integrated multi-omics signature comprising selected bacterial taxa and metabolites. Several key features, including *Prevotella*, *Lachnoanaerobaculum*, urea, valine, and 3-HBA, were consistently identified by both DIABLO and the pairwise correlation analyses. The concordant findings from these complementary analytical approaches increase confidence in the robustness of the identified multi-omics signature, suggesting that these features consistently contribute to the integrated microbiome–metabolome profile rather than representing isolated pairwise associations. Furthermore, the coordinated relationships visualized by the DIABLO Circos network provide additional support for an integrated microbiome–metabolome signature associated with ALMS and BBS, while remaining consistent with the exploratory nature of the study and without implying direct mechanistic relationships between individual taxa and metabolites.

The present study has several limitations. First, the relatively small cohort size reflects the rarity of ALMS and BBS and limits the statistical power of the analyses. Consequently, the findings should be considered exploratory and hypothesis-generating, and require validation in larger, independent cohorts. Second, the cross-sectional design does not allow conclusions about causality or the temporal relationships between microbial and metabolic alterations. Furthermore, the integration analyses were based on previously generated microbiome and metabolomic datasets and were not supported by functional validation experiments. Therefore, the identified microbiome–metabolome associations should be interpreted as coordinated patterns rather than evidence of direct biological interactions or causal mechanisms. Another limitation concerns the taxonomic resolution of the microbiome analysis. Because 16S rRNA gene sequencing generally provides genus-level rather than species-level identification, bacteria within the same genus may differ substantially in their metabolic capabilities. Moreover, given the large number of bacterial genera identified, the discussion focused primarily on broader phylogenetic groups (phyla) to facilitate interpretation of overall biological patterns, rather than emphasizing individual genus-level associations. Consequently, the observed microbiome–metabolome relationships should be interpreted cautiously, as they do not establish functional interactions or causal relationships and require validation using higher-resolution taxonomic approaches, such as shotgun metagenomic sequencing. Finally, although complementary analytical approaches, including pairwise correlation analysis, MCIA, and DIABLO, consistently identified coordinated microbiome–metabolome signatures, these findings should be interpreted within the study’s exploratory framework. Future studies incorporating longitudinal designs, larger patient cohorts, species-level microbiome profiling, and functional multi-omics approaches will be essential to validate these signatures and clarify their biological significance in ALMS and BBS.

## Conclusion

Our integrated profiling of the oral microbiome alongside the salivary and GCF metabolomes in patients with ALMS and BBS revealed distinct microbiome–metabolome association patterns linked to disease-related metabolic alterations and oral microbial reorganization. Elevated levels of fatty acids, BCAAs, and AAAs were associated with shifts in the abundance of selected anaerobic and metabolically relevant taxa, including *Veillonella*, *Tannerella*, *Leptotrichia*, and *Selenomonas*, whereas taxa commonly associated with oral ecological balance, including *Haemophilus*, *Neisseria*, and *Schaalia*, were reduced. Associations involving TCA cycle metabolites and myo-inositol further indicate coordinated microbiome–metabolome relationships that may reflect altered substrate utilization and ecological adaptation within the oral environment rather than direct microbial co-metabolism.

Notably, microbiome–metabolome associations differed between GCF and saliva, suggesting that saliva may preferentially reflect broader systemic metabolic alterations, whereas GCF may offer greater sensitivity to local periodontal and immunometabolic conditions. Pairwise correlation analyses identified disease-associated relationships between bacterial taxa and metabolites, while complementary multi-omics integration using MCIA and DIABLO showed that these associations were part of a coordinated cross-omics structure and identified a robust multi-omics signature shared across the oral microbiome, salivary metabolome, and GCF metabolome.

Overall, our findings demonstrate the value of combining correlation-based analyses with integrative multi-omics approaches to characterize coordinated microbiome–metabolome alterations in rare ciliopathies. Although the present study does not establish causal or mechanistic relationships, it provides a framework for future longitudinal and functional studies to elucidate host–microbiome interactions and microbiome–metabolome relationships, and for further evaluation of saliva and GCF as complementary, noninvasive sources of biomarkers of metabolic dysregulation.

## Ethical Approval

The study protocol was approved by the University Bioethics Committee at the Medical University of Lodz, Poland (RNN/216/23/KE). Written informed consent was obtained from all participants and/or their parents or legal guardians prior to inclusion in the study. Detailed information on ethical approval and informed consent procedures has been reported previously [2,8].

## Data Availability

The datasets generated and analyzed in this study are not publicly available because they contain data from a small cohort of patients with rare genetic disorders and are subject to ethical and privacy restrictions. The datasets are available from the corresponding author upon reasonable request.
