# Supplementary material for: Integrated Oral Microbiome and Metabolome Profiling Identifies Disease-Associated Multi-Omics Signatures in Alström and Bardet–Biedl Syndromes
Source: Comput Struct Biotechnol J. 2026 Sep 3;35(1):0211. doi: 10.34133/csbj.0211 (PMC13538939; doi:10.34133/csbj.0211)
Supplement: Supplementary file 1 [file csbj.0211.f1.zip › Supplementary Materials.docx]

Figure S1. Cumulative variance explained by MCIA components.

**Figure S2.** Variables with the highest absolute contributions to (a) the first and (b) the second MCIA component across the integrated omics datasets. Bar colors indicate the omics block from which each variable originates (GCF metabolome, salivary metabolome, or oral microbiome).

**Figure S3.** Stability of feature selection across repeated 80% subsampling iterations. Bars indicate the selection frequency of individual microbial taxa and metabolites retained by the DIABLO model within each omics block. The dashed line indicates the predefined stability threshold.

Table S1. Complete Spearman's correlation matrix between oral bacterial genera and metabolites in gingival crevicular fluid (GCF). Statistically significant correlations are indicated as *p* < 0.05 (*), *p* < 0.01 (), and *p* < 0.001 (*).

Table S2. Complete Spearman's correlation matrix between oral bacterial genera and metabolites in saliva. Statistically significant correlations are indicated as *p* < 0.05 (*), *p* < 0.01 (), and *p* < 0.001 (*).

**Table S3.** PERMANOVA results for the DIABLO latent components across the microbiome, salivary metabolome and GCF metabolome datasets.

| **Block** | **R2** | **F** | ***p*** |
| --- | --- | --- | --- |
| **Microbiome** | 0.243 | 12.39 | < 0.001 |
| **Saliva** | 0.319 | 18.06 | < 0.001 |
| **GCF** | 0.407 | 26.46 | < 0.001 |
