# Supplementary material for: Integrated Oral Microbiome and Metabolome Profiling Identifies Disease-Associated Multi-Omics Signatures in Alström and Bardet–Biedl Syndromes
Source: Comput Struct Biotechnol J. 2026 Sep 3;35(1):0211. doi: 10.34133/csbj.0211 (PMC13538939; doi:10.34133/csbj.0211)
Supplement: Supplementary file 1 [file csbj.0211.f1.zip › Table S3.docx]

**Table S3.** PERMANOVA results for the DIABLO latent components across the microbiome, salivary metabolome and GCF metabolome datasets.

| **Block** | **R2** | **F** | ***p*** |
| --- | --- | --- | --- |
| **Microbiome** | **0.243** | **12.39** | **< 0.001** |
| **Saliva** | **0.319** | **18.06** | **< 0.001** |
| **GCF** | **0.407** | **26.46** | **< 0.001** |
